# Observation of *Zitterbewegung* in photonic microcavities

**DOI:** 10.1038/s41377-023-01162-x

**Published:** 2023-05-23

**Authors:** Seth Lovett, Paul M. Walker, Alexey Osipov, Alexey Yulin, Pooja Uday Naik, Charles E. Whittaker, Ivan A. Shelykh, Maurice S. Skolnick, Dmitry N. Krizhanovskii

**Affiliations:** 1 Department of Physics and Astronomy, University of Sheffield, S3 7RH Sheffield, UK; 2 Department of Physics and Technology, ITMO University, St. Petersburg, 197101 Russia; 3 Science Institute, University of Iceland, Dunhagi 3, IS-107 Reykjavik, Iceland

**Keywords:** Polaritons, Photonic crystals

## Abstract

We present and experimentally study the effects of the photonic spin–orbit coupling on the real space propagation of polariton wavepackets in planar semiconductor microcavities and polaritonic analogues of graphene. In particular, we demonstrate the appearance of an analogue *Zitterbewegung* effect, a term which translates as ‘trembling motion’ in English, which was originally proposed for relativistic Dirac electrons and consisted of the oscillations of the centre of mass of a wavepacket in the direction perpendicular to its propagation. For a planar microcavity, we observe regular *Zitterbewegung* oscillations whose amplitude and period depend on the wavevector of the polaritons. We then extend these results to a honeycomb lattice of coupled microcavity resonators. Compared to the planar cavity, such lattices are inherently more tuneable and versatile, allowing simulation of the Hamiltonians of a wide range of important physical systems. We observe an oscillation pattern related to the presence of the spin-split Dirac cones in the dispersion. In both cases, the experimentally observed oscillations are in good agreement with theoretical modelling and independently measured bandstructure parameters, providing strong evidence for the observation of *Zitterbewegung*.

## Introduction

The study of analogues to effects appearing in the domain of high energy physics is among the trends of modern condensed matter physics. In this connection, Fabry–Perot optical microcavities, structures where the internal spinor wavefunction can be directly imaged via photon tunnelling through the mirrors, are of particular interest. Such cavities generally support ballistic propagation of two polarisation states of light and have an inherent polarisation–wavevector coupling, the TE–TM splitting, which is an equivalent of spin–orbit coupling (SOC)^1^. They may also support birefringent polarisation splitting, all of which features have allowed observation of a range of important physical effects such as optical spin-Hall effect^2^, the emergence of monopoles^3^ and the onset of the non-Abelian gauge fields^4,5^. In semiconductor microcavities, quantum wells (QWs) can be added, resulting in the formation of composite part-light part-matter quasiparticles called exciton–polaritons. Their unique properties, related to the extremely small effective mass (about five orders of magnitude less than the mass of a free electron), high sensitivity to magnetic fields, giant nonlinear optical interactions, and possibility for optical amplification, have allowed the demonstration of optical condensates with macroscopically large coherence length (in the mm scale)^6^, formation of acoustic black holes and Hawking effect^7^, observation of anomalous Hall drift^8^, and many other effects. In this work, we use such semiconductor microcavities but focus on the properties coming from the photonic constituent of polaritons, and so we demonstrate the relevant fundamental principles using highly photonic (*>*98%) polaritons.

Furthermore, there has been much recent work on engineering the bandstructure of microcavities by imposing a laterally varying optical potential^9–11^. This has allowed the study of flat bands in Lieb lattice potentials^10^, topological physics^12–14^ and engineering of Dresselhaus SOC for photons^15,16^. Such lattices, as well as being substantially more tuneable via the added in-plane degrees of freedom, can be used to build photonic analogues of a wide variety of physically important Hamiltonians based on Bose-Hubbard type models^17^. Combined with the other favourable properties of polaritons discussed above, this creates a very wide perspective for polaritonic simulation.

One of the textbook examples of quantum relativistic effects is *Zitterbewegung*. It consists of an oscillatory motion of a propagating wavepacket transverse to its ballistic trajectory despite the absence of transverse forces^18^. It was first predicted by Schrödinger for the motion of free electrons governed by the Dirac equation^19^ and appears due to interference between positive and negative energy states of a spinor (two-component) system, enabled by the coupling of internal (spin) and external (momentum) degrees of freedom.

In addition to free relativistic electrons, the effect is predicted for electrons in crystals with Rashba and Dresselhaus SOC^20–22^. The predicted high frequency and low amplitude of the oscillations for the vacuum case, and the difficulty of observing single electrons in solids, make experimental observation highly challenging^18^. This has led to a search for analogous systems in which the observation can be made. Gerrtisma et al. ^23^ performed a quantum simulation of the Dirac equation using a single trapped ion. Measurement of the transverse position was made indirectly since most observables cannot be directly measured in ion trap experiments. High-frequency oscillating currents were also observed in the motion of spin-polarised electrons in a doped semiconductor device^24^. In optics, the *Zitterbewegung* was observed in arrays of coupled waveguides where the internal degree of freedom mimicking spin was introduced by having two slightly different waveguides per unit cell^25^. In that case, the energy separation of positive and negative components was fixed by the geometry of the waveguides rather than being continuously tuneable. Thus different lattices had to be used to examine different parameters. Furthermore, the *Zitterbewegung* had to be detected indirectly using the fluorescence induced by the intensity of the light inside the waveguides. Microcavities, by contrast, allow direct imaging and excitation of the internal wavefunction and addressing of different energy separations using pump laser incidence angle and frequency.

Whereas *Zitterbewegung* is usually understood as an oscillation occurring without external forces, it is worth noting that zig–zag oscillations have also been observed in microcavities with a transverse trapping potential^26^. A possible explanation in terms of *Zitterbewegung* was suggested^27^ but still awaits confirmation through detailed analysis and comparison with theory to rule out the effects of the transverse potential. Similarly, it was recently shown that polariton SOC contributes to the periodicity of transverse oscillations of condensate in an etched ring trap, alongside the contribution from condensate motion in the transverse trapping potential^28^. Since the SOC underpins *Zitterbewegung* in planar structures, its contribution may be interpreted as a manifestation of *Zitterbewegung* in the ring traps. However, no direct observation of *Zitterbewegung* was possible. In general, oscillations in structures with transverse trapping potentials are hard to attribute to *Zitterbewegung* since there are alternative explanations, such as interference between multiple transverse modes^29^.

So far, *Zitterbewegung* has not been directly observed in the microcavity structures where polaritons can be formed and which allow the wide range of optical analogues discussed above, although it has recently been theoretically predicted in both planar microcavities^30^ and honeycomb microcavity lattices at wavevectors close to the Dirac point^16^. In this work, we bridge this gap between theory and experiment and report the observation of *Zitterbewegung* in both types of structures. For the planar cavity, we demonstrate tuning of the *Zitterbewegung* period by varying the incidence angle and propagation direction in the birefringent cavity. In the case of wavevectors near the Dirac point in a honeycomb lattice, the ability to engineer the bandstructure allows observation of smaller period *Zitterbewegung* while retaining an observable amplitude. The *Zitterbewegung* essentially arises from interferences between the two components of the spinor wavefunction. The propagating beam has finite spatial width and hence finite width in momentum (angular) space. Since the polarisation states depend on the momentum through the spin–orbit coupling, different angular components of the beam have different relative amplitudes of the spinor components, and these can evolve with propagation. As discussed above, we experimentally demonstrate the essential principles of the effect in the highly photonic regime, but polaritons can be made more excitonic by simple tuning of the photon–exciton detuning, which opens up wider perspectives.

## Results

**Fig. 1:**
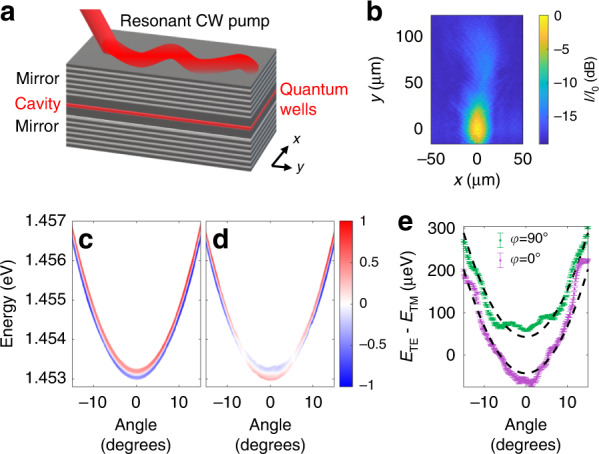
*Zitterbewegung* in planar cavities. **a** Schematic of the planar cavity structure resonantly excited at one point with the photons propagating away along the cavity. **b** Intensity of the photon field in the cavity when excited resonantly vs. *x* and *y*. The colour scale gives the intensity *I* relative to the peak intensity $I_0$ in decibel units. The zero of the *y*-axis is defined as the point of peak intensity vs. *y*. **c** Angle and polarisation resolved photoluminescence spectrum showing the dispersion relation $E\left( {k_y} \right)$ at a fixed $k_x = 0$ for the case where the birefringent crystal principle axis $y^\prime$ ($\varphi = 90$°, see Supplementary Section 1) is parallel to the direction *y* along which the polaritons are injected in the resonant excitation experiment. **d** As panel (c) but for the case where $x^\prime$ is parallel to *y* ($\varphi = 0$°). In (c) and (d) the colour scale indicates the polarisation degree $\left( {I_x - I_y} \right)/\left( {I_x + I_y} \right)$ with red indicating *x* polarisation and blue indicating *y* polarisation ($I_x$ and $I_y$ are the intensities in the *x* and *y* polarisations). Points with total intensity $\left( {I_x + I_y} \right)$ less than 0.20 of the peak have been set to white since the polarisation degree is not well defined for low intensities. **e** The energy splitting between the TE and TM polarisations for the two values of *φ*. Points show the values extracted by fitting Lorentzian peaks to the data in panels (**c**, **d**). Dashed black curves are the fits described in the main text

We begin with the case of the planar microcavity. In these structures, a two-wavelength thick cavity layer is enclosed between two Bragg mirrors (periodically repeating stacks of quarter-wave layers of two different materials), as illustrated in the schematic in Fig. 1a. In our case, the cavity is made of GaAs, the mirror materials are GaAs and Al_0.85_Ga_0.15_As, and the structure was grown by molecular beam epitaxy. Three In_0.04_Ga_0.96_As QWs are embedded in the cavity. The energy detuning between the QW excitons and the cavity photons is more than 20 meV.

The polarisation and angle-dependent reflection of the cavity mirrors leads to a wavenumber-dependent energy splitting (TE–TM splitting) of the cavity modes having electric and magnetic fields transverse to the wavevector. This combines with a slight optical birefringence^31–33^, resulting in a complicated splitting between linear polarisation states. For small in-plane wavevector components, the Hamiltonian of the system in the basis of circularly polarised states reads^33^

1 $$\hat H = \left( {\begin{array}{*{20}{c}} {\frac{{\hbar ^2k^2}}{{2m}}} & {\frac{{\Omega }}{2} - \beta \left( {k_x^\prime - ik_y^\prime } \right)^2} \\ {\frac{{\Omega }}{2} - \beta \left( {k_x^\prime + ik_y^\prime } \right)^2} & {\frac{{\hbar ^2k^2}}{{2m}}} \end{array}} \right)$$

where *m* is the effective mass of the polaritons, $k_x^\prime$ and $k_y^\prime$ are the in-plane wavevector components of the photons in the sample reference frame where *x'* is the fast axis (see Supplementary Fig. 1), $k^2 = k_x^{\prime 2} + k_y^{\prime 2}$, and parameters Ω and *β* describe the values of the k-independent optical birefringence and TE–TM splitting, respectively. The parameter *β* is related to the difference of the longitudinal and transverse masses of the photons *m*_*l*_ and *m*_*t*_ as^34^

2 $$\beta = \frac{{\hbar ^2}}{4}\left( {\frac{1}{{m_t}} - \frac{1}{{m_l}}} \right)$$

To clarify the notation, *m*_*t*_ and *m*_*l*_ are the masses of the TE and TM polarised photons. Note that we define *β* with the opposite sign compared to the definitions in refs. ^33,34^, but this does not affect the physics. The corresponding dispersions of the two photon branches split in linear polarisations read

3 $$E_ \pm = \frac{{\hbar ^2k^2}}{{2m}} \pm \sqrt {\beta ^2k^4 - \beta {{\Omega }}k^2\cos 2\varphi + \frac{{{{\Omega }}^2}}{4}}$$

where *φ* is the in-plane angle between the wavevector ***k****'* and the *x'*-axis of the crystal. With these definitions, the energy of the TE polarised mode (electric field perpendicular to ***k****'*) increases faster with *k* than the TM mode for positive *β*, and at *k* = 0 the mode polarised along *x'* has higher energy for positive Ω. Note, that the combination of birefringence and TE–TM splitting leads to a clear in-plane anisotropy of the dispersions, which cross for *φ* = 0 at $k = \sqrt {{{\Omega }}/\left( {2\beta } \right)}$. This will now be seen experimentally as we present the basic characterisation of the sample.

The experiments in this paper were performed at approximately 10 K temperature in a continuous-flow cold-finger cryostat. The energy vs. wavevector dispersion relations *E*(*k*_*y*_) at *k*_*x*_ = 0 was measured by angle and polarisation resolved photoluminescence (PL) spectroscopy and can be seen in Fig. 1c, d. Note that *x* and *y* are coordinates in the laboratory reference frame (see Fig. 1a). In both figures, the angle *θ* on the horizontal axis gives the wavevector $k_y = k_0\sin \theta$, $k_x = 0$, where $k_0 = 2\pi /\lambda$. Fig. 1c shows the case where the sample is rotated such that ${{{\boldsymbol{k}}}} = k_y{{{\hat{\boldsymbol y}}}}$ is parallel to $y^\prime$ (*φ*=90°). Two branches with different polarisations are visible. As expected, there is a splitting at *k* = 0 resulting from the birefringence and the splitting increases with *k* due to the TE–TM splitting. Fig. 1d shows the case where the sample is rotated such that ${{{\boldsymbol{k}}}} = k_y{{{\hat{\boldsymbol y}}}}$ is parallel to $x^\prime$ (*φ* = 0°). In this case, the dispersions cross at 5.7° (*k* = 0.73 µm^−1^), again as expected. Note that at the crossing point, the polarisation degree is low because the contributions from the two branches have similar intensity but are not coherent with one another owing to the spontaneous nature of photoluminescence emission. For each measured dispersion, we extract the energy at the peak intensity for each angle and for each polarisation and fit the resulting energy vs. angle curves to find the parameters describing the sample. We find *Ω* = 43 ± 19 µeV, *β* = 33.6 ± 3.5 µeV µm^2^. The energy at *k* = 0 (averaged between the two dispersions) is 1.4531 eV and $\hbar ^2/(2m) =$ 947.5 µeV µm^2^.

We now proceed to measure the *Zitterbewegung* effect in the sample. We excited the cavity resonantly using a tuneable continuous-wave Ti:Sapphire laser, as illustrated in Fig. 1a. The laser energy and incident angle were set to match points on the dispersion allowing efficient injection of photons into the cavity. The laser spot incident on the sample was circular with full-width-at-half-maximum (FWHM) 15 µm. The size of the Fourier transform of this spot, e.g. its size vs. wavenumber *k*, was sufficient to efficiently excite both polarisation branches. The excitation polarisation was circular to ensure equal excitation of both branches. The light was collected from the opposite side of the sample to the excitation (transmission geometry), and the total intensity was recorded by a thermo-electrically cooled CCD camera. An example of the recorded intensity pattern is shown in Fig. 1b. The highest intensity part peaking at *y* = 0 µm is related to the incident laser spot. Since we excite at a finite angle where the group velocity (slope of the dispersion) is finite, the photons in the cavity propagate away from the excitation spot in the positive *y* direction, decaying by emitting photons through the Bragg mirrors towards the detector. For any value of *y*, we can take a slice along *x* and find the value of *x* at which the intensity is maximum. To minimise the effects of the scatter of the data points, it is better to define the centre of intensity according to

4 $$x_c\left( y \right) = \frac{{\mathop {\int }\nolimits_{ - \infty }^\infty x \cdot I\left( {x,y} \right) \cdot dx}}{{\mathop {\int }\nolimits_{ - \infty }^\infty I\left( {x,y} \right) \cdot dx}}$$

**Fig. 2:**
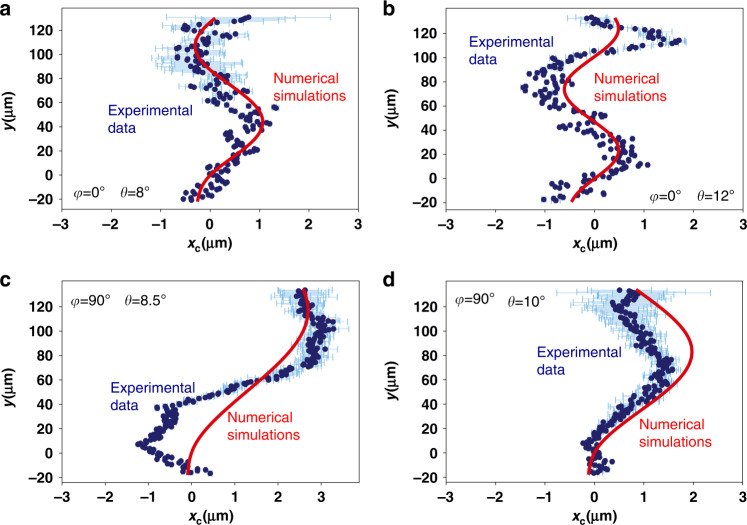
Centre of intensity *x*_*c*_ vs. *y* for resonant excitation at two different angles and for two orientations of the sample with respect to the propagation direction *y*. **a**, **b** The case where the principle axis $y^\prime$ is aligned along *y* and corresponds to the dispersion curve in Fig. 1c. **c**, **d** The case where the principle axis $x^\prime$ is aligned along *y* and corresponds to the dispersion curve in Fig. 1d. Blue points correspond to the experimental data, the red solid line gives the theoretical fit. The zero of the *y*-axis is defined as the point of peak intensity vs*. y*. The zero of the *x*-axis is defined such that the theoretical $x_c\left( {y = 0} \right) = 0$. Error bars are plotted between $\pm \sigma$ where *σ* is the standard deviation calculated as discussed in Supplementary Section 4. Error bars smaller than the data point symbols are not plotted

In Fig. 2, we plot $x_c(y)$ for different incidence angles and orientations of the sample. Fig. 2a, b shows the cases for 8° and 12° angles of incidence, respectively and where we inject light along the $y^\prime$-axis of the cavity (*φ* = 0). The blue points give the experimentally extracted trajectory of the wavepacket. Clear oscillations of $x_c$ are visible with increasing distance *y* away from the excitation spot. We discuss sources of uncertainty in $x_c$ in Supplementary section 4. For the *Zitterbewegung* effect, the period of the oscillation *L* corresponds to the frequency separation of the positive and negative branches and is expected to vary with excitation angle^30^. Qualitatively, at the larger angles, we expect higher frequency (shorter period) oscillations as the TE–TM splitting between the branches increases (see Fig. 1e). As expected, we see in the experiment that the period becomes shorter at higher angles. We simulated the expected trajectory of the wavepackets using a model of the evolution of the spinor wavefunction accounting for both TE–TM splitting and birefringence (see Supplementary Section 1). The red curves in Fig. 2a, b shows the theoretical evolution of $x_c$ vs. *y*. The parameters *Ω* = 28.8 µeV µm^2^ and *β* = 32.45 µeV µm^2^ for the simulation were obtained by fitting the experimental oscillations. Both the period and amplitude of the simulated trajectories are in excellent agreement with the experimental points. Importantly, the values of *β* and *Ω* found from fitting the oscillations are in good agreement with the values found independently from fitting the dispersion relations (*β* agrees within 0.33 of the uncertainty, *Ω* agrees within 0.75 of the uncertainty), as discussed above. We then rotated the sample and measured the oscillations for the case where we injected light along the $x^\prime$-axis of the cavity (*φ* = 90°). As discussed above, this changes the nature of the dispersion, in particular, the splitting between the two polarisation branches for a given angle. The data is shown in Fig. 2c, d for incidence angles 8.5° and 10°, respectively. We used exactly the same parameters as in the *φ* = 0° case to simulate the trajectory of the light and obtain semi-quantitative agreement between the experimental points and the theory without further fitting. The agreement of the amplitude is slightly less good than in the *φ* = 0° case due to proximity to the crossing of the two polarisation branches (Fig. 1d) at 5.7°. At this point, there is no splitting, and the period becomes singular, with the oscillations being correspondingly more sensitive to the exact parameters at angles close to the singular point.

Finally, the *Zitterbewegung* effect arises due to interference between the two polarisation components, e.g. it is fundamentally a spinor effect. From theory, we expect that the oscillations in the total intensity pattern should disappear if only one polarisation branch is excited. We tested this in two ways, described in more detail in Supplementary Section 2. First, we tuned the excitation angle so that one branch was preferentially excited and saw that the amplitude of the oscillations gradually reduced and disappeared. Second, we excited the system with light linearly polarised parallel to one polarisation branch and perpendicular to the other and did not detect oscillations.

**Fig. 3:**
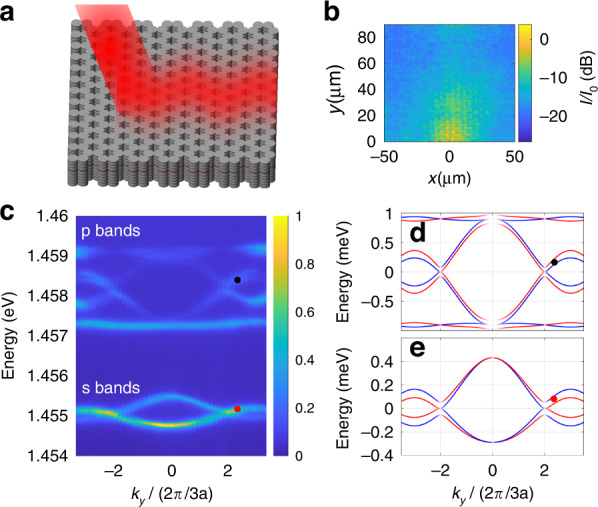
*Zitterbewegung* in honeycomb lattice. **a** Schematic of the honeycomb lattice structure resonantly excited at one point with the photons propagating away along the structure. **b** Intensity vs. *x* and *y* recorded for excitation at the black point in panel (c). Colour scale gives the intensity *I* relative to the peak intensity $I_0$ in decibel units. The zero of the *y*-axis is defined as the edge of the lattice where the pump spot is incident. **c** Dispersion relation of the honeycomb lattice measured by angle-resolved photoluminescence spectroscopy without polarisation resolution. Colour scale gives the intensity relative to the peak value. The red and black points mark the excitation energies and corresponding incidence wavevectors at which the *Zitterbewegung* effect is studied. **d**, **e** Dispersion of the p-bands (**d**) and s-bands (**e**) calculated by a tight binding model with parameters from ref. ^16^. Red and blue curves represent states linearly polarised along *x* and *y*, respectively

The planar microcavity, with its simple structure and tuneability through excitation angle, is a good platform for proof of principle demonstrations. Lattices formed by etching the microcavity to introduce a lateral pattern allow a wide variety of physically important band structures to be simulated and are highly tunable via lateral patterning. They are thus an important complementary platform which opens up a wide perspective for future studies. We, therefore, studied *Zitterbewegung* in a honeycomb lattice, a photonic analogue of graphene. The lattice is formed by etching the planar cavity into air post cavities. These so-called pillar microcavities support discrete energy states localised in all three spatial dimensions. By overlapping neighbouring pillars (see Fig. 3), we allow photons to tunnel from one pillar to another, allowing the discrete states to hybridise and form energy bands. The structure we study here is a honeycomb lattice, which is a triangular lattice with 2 pillars per unit cell. The diameter of the pillars is 3 µm, the centre-to-centre spacing of adjacent pillars is *d* = 2.8 µm, the lattice periodicity is $a = d\sqrt 3$, the lattice vectors are $a\left( {\sqrt 3 /2, \pm 1/2} \right)$ and the reciprocal lattice vectors are $(2\pi /a)\left( {1/\sqrt 3 , \pm 1} \right)$.

In Fig. 3a, we show a schematic diagram of the honeycomb lattice structure being excited by the laser beam, resulting in the propagation of the light in the cavity accompanied by transverse oscillations. An example of an experimentally measured intensity pattern is shown in Fig. 3b and is equivalent to the pattern seen in the planar case (Fig. 1b), except that it results from the propagation of photons in the bands of the lattice rather than those of the planar cavity. The band structure of the lattice can be seen in the angle-resolved photoluminescence spectrum of Fig. 3c. Two distinct sets of bands separated by a band gap can be seen. The lowest set of bands, labelled ’s bands’, are composed of the lowest energy states of the individual pillars, resembling lowest order Hermite–Gauss modes^16^. The next highest set of bands, labelled ‘p bands’, is composed of the first-order Hermite–Gaussian modes of the individual pillars. For the purpose of this work, the essential difference between the two sets of bands is that the polarisation-dependent tunnelling rates from pillar to pillar are different, resulting in different splittings and different group velocities (Fermi velocity) close to the Dirac points (at $k_x = 0$, $k_y = 4\pi /\left( {3a} \right)$). We, therefore, expect different *Zitterbewegung* periods and amplitude close to the Dirac points in either band.

The red and black dots in Fig. 3c mark the energy and wavevector at which we resonantly excite the bands to measure the *Zitterbewegung*. As in the planar case, we equally excite the two bands. More detailed plots of the band structure are given in Fig. 3d, e for the p and s bands, respectively. These are obtained from a tight binding model using parameters from ref. ^16^, where the same lattice was studied extensively, a detailed analysis of polarisation splitting was performed, and the model was fit to the dispersion. The red and blue colour of the lines denote *x* and *y* polarised waves, respectively, and the dots (red for s-bands and black for p-bands) denote the energy and wavevector of excitation, slightly detuned from the Dirac points. Around these excitation points, the energy splitting Δ*E* between the bands is 65 µeV for the s-bands and 96 µeV for the p-bands. The wavenumber splitting ${{\Delta }}k_y$ between the two bands at the fixed laser energy is 0.49 $\times 2\pi /\left( {3a} \right)$ for the s-bands and 0.25 $\times 2\pi /\left( {3a} \right)$ for the p-bands.

**Fig. 4:**
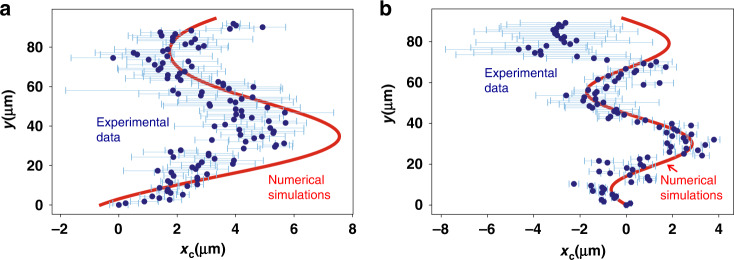
Centre of intensity *x*_*c*_ vs. propagation distance *y* for resonant excitation at two different angles. Blue points give the experimental data. A red solid line gives the theoretical fit. **a** Case for excitation of the p-band states, corresponding to the black circle in Fig. 3d. **b** Case for excitation of the s-band states, corresponding to the red circle in Fig. 3e. The zero of the *y*-axis is defined as the edge of the lattice where the pump spot is incident

As in the planar case, we use Eq. 4 to extract $x_c$ vs. *y* from the intensity pattern of the propagating photons. Fig. 4a, b shows the extracted trajectories of the photons for the p and s bands, respectively. Oscillations of $x_c$ are visible, and we see that the period of oscillation is shorter for the s-bands. The period is expected to scale as $L = 2\pi /{{\Delta }}k_y$. Therefore the shorter period for the s-band is consistent with the larger ${{\Delta }}k_y$ near the excitation point for the s-band. We calculated the theoretical trajectory for the photons in a similar way as for the planar case but using evolution equations for states near the Dirac point of the honeycomb lattice^16,35^ (see Supplementary Section 3). Parameters for the modelling were taken from ref. ^16^, where the same lattice was studied extensively, and the dispersion relation was fitted. Semi-quantitative agreement is obtained between the theory and experiment.

## Discussion

In the experiments on the planar cavity, clear oscillations of the centre of mass with increasing propagation distance were observed. The oscillation period decreased with increasing splitting between the branches, and the oscillations disappeared when only one branch was excited, as expected. The expected trajectory of the centre of mass was also calculated using a model of the evolution of the spinor wavefunction. The model reproduced the experimental period and amplitude using parameters in good agreement with those obtained independently from the dispersion curve measurements. The amplitude of *Zitterbewegung* oscillations is, in general, a complicated function of sample and excitation parameters but, to a good approximation, is governed by the experimentally chosen excitation angle^30^ and, unlike the period, is independent of the TE–TM splitting and birefringence parameters *β* and *Ω*. The fact that both the observed period and amplitude agree well with the model provides strong evidence that the oscillations we observe experimentally are indeed the real transverse oscillations of the wavepacket in the cavity, which is the *Zitterbewegung*. We also studied *Zitterbewegung* in honeycomb lattices, where clear oscillations were once again observed with period and amplitude in good agreement with the numerical modelling.

In summary, we experimentally demonstrate *Zitterbewegung* in planar microcavity and microcavity honeycomb lattices. Unlike previous demonstrations, the Fabry–Perot cavity design allows direct visualisation of the complex spinor field inside the device. Lattice structures allow the building of photonic analogues to important physical systems, such as the photonic graphene we study here. In exciton-polariton lattices, identical to our structure, apart from smaller energy detuning between photons and quantum well exciton, interparticle interactions and high sensitivity to magnetic fields can easily be added. This work then opens the door to studying a very wide class of photonic analogues of relativistic systems with particle interactions, time-reversal symmetry breaking and dissipative effects.

## Supplementary information

Supplementary-Observation of Zitterbewegung in photonic microcavities

## Data Availability

The data supporting the findings of this study are freely available in the University of Sheffield repository with the identifier 10.15131/shef.data.22303282.
