# Supplementary material for: Observation of Zitterbewegung in photonic microcavities
Source: Light Sci Appl. 2023 May 23;12:126. doi: 10.1038/s41377-023-01162-x (PMC10205819; doi:10.1038/s41377-023-01162-x)
Supplement: Supplementary file 1 — Supplementary-Observation of Zitterbewegung in photonic microcavities [file 41377_2023_1162_MOESM1_ESM.pdf]

Observation of *Zitterbewegung* in photonic microcavities

Seth Lovett,[1] Paul M. Walker,[1,*] Alexey Osipov,[2] Alexey Yulin,[2] Pooja Uday Naik,[1] Charles E. Whittaker,[1] Ivan A. Shelykh,[2,3] Maurice S. Skolnick,[1] and Dmitry N. Krizhanovskii[1]

[1]*Department of Physics and Astronomy, University of Sheffield, S3 7RH, Sheffield, UK*
[2]*Department of Physics and Technology, ITMO University, St. Petersburg, 197101, Russia*
[3]*Science Institute, University of Iceland, Dunhagi 3, IS-107, Reykjavik, Iceland*

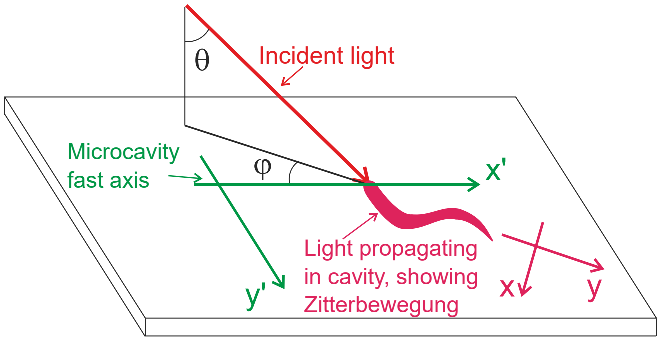

FIG. S1. The sketch of the system showing the direction of the incident light wave vector (red arrow), its projection on the waveguide plane, the orientation of the principal axes of the waveguide (green). Propagation of the guided waves is illustrated schematically by magenta color. The laboratory frame axes x and y, shown by magenta lines, are oriented along (y) and orthogonal (x) to the light incidence plane. The angle of incidence is denoted as θ and the angle between y and the principal axis x' is φ.

I. MODELLING OF *ZITTERBEWEGUNG* IN PLANAR MICROCAVITY

In this section we shed the light on the technical details of the numerical simulation of the *Zitterbewegung*. We start with the case of the cavity formed by two Bragg mirrors separated by a dielectric layer. The mirrors have finite transparency and thus can be excited by a light beam at some incidence angle θ, see Fig. S1 showing schematically the experimental setup. To reproduce the experimental data we need to take into account that the the phase of the complex reflection coefficient of the mirrors depends on the polarization, i.e. there is TE-TM splitting of the guided waves.

The dielectric layers in the experiment are birefrigent and we account for this in our numerical simulations. Because of the anisotropy we need to characterize the orientation of the light incidence plane with respect to the principal axes of the structure. For this we introduce the angle φ as the angle between the light incidence plane (the $y-z$ plane) and one of the principal axes (the fast axis) denoted as x', see Fig. S1.

The cavity is thin (having thickness of only two wavelengths) and so only the two longitudinal modes lowest in frequency (one for each of two orthogonal polarisations) have to be taken into account. Then to describe the evolution of the electromagnetic field in the cavity it is convenient to use the slow varying amplitude approach and characterize the field by complex amplitudes of two modes having different polarizations. In the basis of circular polarizations the equations for the amplitudes read

$$\begin{aligned} i\hbar\partial_t\psi_\pm + \frac{\hbar^2}{2m}\nabla^2\psi_\pm + i\gamma\psi_\pm + \frac{\Omega}{2}\psi_\mp \\ +\beta(\partial_{x'} \pm i\partial_{y'})^2\psi_\mp = P_\pm f(r)e^{-i\Delta t+i(\boldsymbol{k'}\cdot\boldsymbol{r'})} \end{aligned} \tag{S1}$$

where $\psi_\pm$ are the slow varying amplitudes of the clock- and counter-clockwise circular polarizations, γ is the losses, Ω is the splitting at $k=0$ caused by birefrigence, β is the TE-TM splitting strength, $P_\pm$ are the pump produced by two polarizations of the incident beam, $f(r)$ is the function describing the spatial aperture of the incident field, Δ is the detuning of the incident light frequency from the minimum energy of the cavity dispersion (see Fig. 1c and 1d in the main text) and $\boldsymbol{k'}$ is the projection of the wave vector of the incident beam on the cavity plane. Experimentally, the transmitted field corresponds to $\psi_\pm$ and we plot the total intensity $|\psi_+|^2 + |\psi_-|^2$ in Fig. 1b in the main text.

To find the parameters of our mathematical model (Eqn. S1) we fit the experimental oscillations in Figs. 2(a) and 2(b) in the main text. The parameters we obtain, and which are used in the simulations below, are $\Omega = 28.8$ µeV·µm^2 and $\beta = 32.45$ µeV·µm^2. We take the parameters $m = 950$ µeV·µm^2 and $\gamma = 34$ µeV from the fitting of the experimental dispersion curves discussed in the main text. The incident light beam has a Gaussian profile $f(r) = e^{-\frac{r^2}{L^2}}$ with the half-width $L = 15$ µm. The incident wavevector in the sample coordinate frame is given by $\boldsymbol{k'} = [\frac{2\pi}{\lambda}\sin(\theta)\cos(\varphi), \frac{2\pi}{\lambda}\sin(\theta)\sin(\varphi)]$.

The stationary solutions of (S1) are calculated by the expansion of the field over the plane wave modes, finding the mode amplitudes and then summing the modes to get the total field. Then, knowing the spatial distribution of the field for fixed frequencies Δ it is easy to calculate the intensity distribution of the total field or in the polarization of interest. To calculate the trajectory $x_c(y)$ we calculate the first moment (expectation) defined in a common way as $x_c(y) = \int Ixdx / \int Idx$ where I is the field intensity.

To observe the *Zitterbewegung* we perform the simulations for a circularly polarized pump with the wavevector

* p.m.walker@sheffield.ac.uk

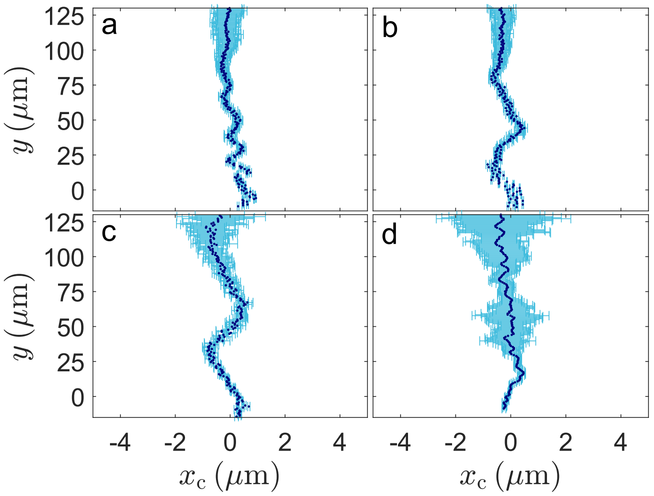

FIG. S2. Center of intensity x_c vs y. (a) Resonant excitation of one branch only using linear polarisation. (b) Resonant excitation of both branches using circular polarisation. For both (a) and (b) the incidence angle was 11 degrees and the laser energy was 1.90 meV above the bottom of the dispersion, equally resonant with both branches. (c) Resonant excitation at 10 degrees and energy 1.57 meV above the bottom of the dispersion and with circular polarisation. (d) Off-resonant excitation at the same energy and polarisation as in (c) but at 11 degrees. Black points are raw experimental data. Red curves are the result of smoothing the data with a 30 micron wide rectangular smoothing window.

$k = \sqrt{\frac{2m\Delta}{\hbar^2}}$ equally detuned from the wave vectors of the eigenmodes of different polarizations. This ensures the nearly equal excitation efficiency for both of the modes. The comparison of the results of the numerical simulations with the experimental data are shown in Fig. 2 in the main text of the paper, for the incident beam directed along the first and the second principle axes of the sample. There is good agreement between the simulated curves and the experimental points for the parameters given above.

II. OFF RESONANCE EXCITATION OF THE CAVITY

Zitterbewegung occurs due to an interference between two polarisation components. The characteristic oscillation of the center of mass x_c of the total intensity distribution is only expected to be observed when both polarisation branches are equally excited. Figures S2(a) and (b) show x_c vs. y for the cases where only one branch was excited and both branches were excited, respectively. This was achieved by setting the polarisation state of the incident laser to either linear or circular respectively. Figure S2(a) shows a small drift of $x_c(y)$ with low amplitude and short period (~ 20 µm) approximate oscillations resulting from a low intensity parasitic signal. The very short period means we can be confident that these are not related to *Zitterbewegung*. By contrast, Figure S2(b) shows much larger amplitude and longer period oscillations consistent with the *Zitterbewegung* effect. We also studied the case where one branch was more strongly excited than the other because we detuned the laser incidence angle away from the midpoint of the two branches. In this case the excitation polarisation was always circular. The angular half width at half maximum of the Fourier transform of the laser spot is 0.7 degrees, and we tuned ~ 1 degree either side of the resonance (e.g. the point where both branches are excited equally) in steps of 0.3 degrees. The position of equal excitation of both branches was estimated by maximising the total transmitted intensity. Figure S2(c) shows the case where both branches are equally excited (10 degrees incidence angle), while Fig. S2(d) shows the case where the excitation laser spot in momentum space overlaps one branch more strongly than the other (11 degrees incidence angle). In both cases the laser energy is the same, 1.57 meV above the bottom of the dispersion. In the case of the off-resonance excitation the lower transmitted intensity and consequently lower signal to noise ratio results in larger error bars. As expected, the oscillations disappear when only branch is strongly excited such that the contribution from interference between the branches is weak.

III. MODELLING OF *ZITTERBEWEGUNG* IN HONEYCOMB LATTICES

The simulation of *Zitterbewegung* in honeycomb lattices was done using the model introduced in[1,2]. The model describes the dynamics of the modes with the wavevectors lying in the vicinity of the K-points of the lattice dispersion relation, where the dispersion has the shape of a Dirac cone. In such lattices the polarisation splitting is dominated by the difference in tunnelling rate for photons with polarisation aligned along or orthogonal to the vector between two pillars[1]. Since the tunnelling vector depends on the photon wavevector this splitting is similar to a TE-TM splitting, though not exactly the same as that in planar structures. The model accounts for this splitting, which lifts the polarization degeneracy of the cone, while it neglects the anisotropy (birefringence) of the dielectric layers. This approach is justified as it was shown in Ref.[2] that the polarisation properties of this sample could be fully described using only the tunnelling-based polarisation splitting. The model is applicable for s and p-states but the parameters have to be fitted separately for each of the cases.

Within the model the equations are written for four field components $\Psi = [\psi_A^+, \psi_B^+, \psi_A^-, \psi_B^-]$, where $\pm$ denotes the polarization and A, B marks the sublattice degree of freedom (different symmetry of the wavefunction over the two sites contained in the elementary cell of the periodic structure). The equations for the evolution of $\psi_{\pm\, A,B}$

read:

$$i\hbar\partial_t\Psi = -i\gamma\Psi + \hbar v_F(-\hat{\sigma}_y\hat{q}_x + \hat{\sigma}_x\hat{q}_y)\Psi + \Delta_{LT}(\hat{s}_y \otimes \hat{\sigma}_y - \hat{s}_x \otimes \hat{\sigma}_x)\Psi + +Pf(r)\exp(-i\omega t + i(\boldsymbol{k}\cdot\boldsymbol{r})), \quad \text{(S2)}$$

where $\boldsymbol{q}$ is the wavevector detuning from the K symmetry point, $\hat{\sigma}_i$ and $\hat{s}_i$ are Pauli matrices acting on sub lattices and polarization degrees of freedom, Δ_{LT} is the TE-TM splitting strength, and v_F is the Fermi velocity. The exact parameters, taken from Ref.[2], are $\hbar v_F = 0.504$ meV·µm, $\Delta_{LT} = 0.027$ meV for the s band and $\hbar v_F = 1.68$ meV·µm, $\Delta_{LT} = -0.0375$ meV for the p band. The external pump contains four components accounting for the pumping of two polarization, each of which has two different symmetries of the field distribution on the elementary cell, so $P = [P_A^+, P_B^+, P_A^-, P_B^-]$

The eigen-energies of the states as function of the momentum are given by the dispersion characteristics

$$E_{up,\pm} = E_0 \pm \Delta_{LT} + \sqrt{\Delta_{LT}^2 + (\hbar v_F \boldsymbol{q})^2} \quad \text{(S3)}$$

for the upper branch and

$$E_{lw,\pm} = E_0 \mp \Delta_{LT} - \sqrt{\Delta_{LT}^2 + (\hbar v_F \boldsymbol{q})^2} \quad \text{(S4)}$$

for the lower branch. One can see that TE-TM splitting transforms the degenerate Dirac cones to a cone with a hyperbolic branch embedded inside the cone.

The Fermi velocity v_F defines the slope of the cone and can be found by fitting the experimental dispersion characteristic in the vicinity of K-point. The same fitting gives the value of TE-TM splitting. The symmetry of the excitation beam in the experiment fixes the structure of the pump $P_A^\pm = P_B^\pm$.

The stationary solutions of equations (S3)-(S4) have been found by the same method as we used for the planar structure. So the field was represented as a series of the plane waves, then the amplitudes of the modes were calculated. Then the trajectory of the center of mass of the light, $x_c(y)$, was calculated in the same way as for the planar structure. To reproduce the experimental results the pump was taken in the form corresponding to linear polarization, calculating the trajectory of propagating light the intensity in a circular polarization was used. The angle of incidence of the coherent pump was chosen to provide equal excitation efficiency for two eigenmodes of the system. The excitation frequencies were chosen as in the experiments.

IV. SOURCES OF UNCERTAINTY IN THE CENTER OF INTENSITY

The main source of random scatter in the data points shown in Figs. 2 and 4 in the main text arises due to noise in the intensity images recorded on our CCD camera. This noise mainly consists of CCD dark counts and read noise. To estimate the error bars we model the contribution of experimental noise to the center of intensity in the following way. The center of intensity is defined by

$$x_c(y) = \frac{\int_{-\infty}^{\infty} x \cdot I(x,y) \cdot dx}{\int_{-\infty}^{\infty} I(x,y) \cdot dx} \quad \text{(S5)}$$

The intensity is recorded on a CCD camera as a number of counts n per pixel as a function of x and y. The number of counts can be written as the sum of two terms: true signal counts n_s and random noise counts n_n. The signal arises due to the light falling on the detector. The noise arises primarily from detector dark counts and read noise. The noise counts in each pixel are modelled as being independent of one another and normally distributed with zero mean and variance σ^2.

The center of intensity then becomes

$$x_c(y) = \frac{\sum_{i=-N}^{N} x n_s + \sum_{i=-N}^{N} x n_n}{\sum_{i=-N}^{N} n_s + \sum_{i=-N}^{N} n_n} \quad \text{(S6)}$$

where the index i runs over the $2N$ pixels in the x direction over which the signal is defined and the corresponding values of x run from $-x_{\max}$ to $x_{\max}$.

We define the total number of signal counts as

$$S = \sum_{i=-N}^{N} n_s. \quad \text{(S7)}$$

The denominator of Eqn. (S6) is normally distributed with mean S and variance $2N\sigma^2$. As we verified numerically (and will describe in a moment), provided that $S \gg \sigma\sqrt{2N}$ (which is always fulfilled in the experiments) the value x_c from Eqn. S6 is approximately normally distributed with variance

$$\sigma_c^2 \approx \frac{2\,(N+1)\,(2N+1)}{6N}\left(\frac{\sigma x_{\max}}{S}\right)^2 \quad \text{(S8)}$$

This formula gives the uncertainty in x_c due to the noise in the data. To plot the error bars in Figs. 2 and 4 in the main text we estimated S from the experimental data by summing the counts over all $2N$ pixels. The value of σ was taken from the experimental data by taking the standard deviation of points at large x, far into the tails of the Gaussian envelope of the propagating beam where the signal is negligible. We then used the value σ_c given by Eqn. (S8) as the error bar size for the figures. Note in this formula that the uncertainty σ_c is proportional to σ/S, in other words it is proportional to the inverse of the signal to noise ratio. In general the signal reduces exponentially with propagation distance

due to the finite lifetime of the photons in the cavity. The noise level remains approximately constant resulting in an exponentially decreasing signal to noise ratio with increasing propagation distance.

To confirm the accuracy of the above method, and of Eqn. (S8), we performed Monte-Carlo simulations in MATLAB. We generated many simulated data sets with all the relevant parameters (such as N,σ,S,x_{max},Gaussian beam shape) equal to those in the experiments. We then calculated x_{c} in the same way as we did for the experimental data and took the standard deviation. These values agreed to within a few percent with the standard deviations obtained from Eqn. (S8) as described above, which validates our method.

As well as the random noise in the CCD counts there could also be small contributions (on the order of a few percent of the main beam intensity) due to stray scattered laser light, small reflections from optics in the beam path, or parasitic laboratory light. Although we did not see obvious evidence of these in the CCD images we cannot rule out that low intensity contributions of this kind could still slightly modify the measured center of mass. Such effects are most likely to affect the center of mass at large propagation distances where the real signal has reduced a lot due to the exponential decay. However, it is not easy to model such effects in order to include them in error bars. Finally, there can also be systematic errors due to imperfections in the incident laser spot shape and focussing, slight deviations from the target excitation polarisation and incidence angle, and other such features which can contribute to the residual discrepancy between the experimental data and the theory. Overall, however, the agreement is good and provides strong evidence that the observed oscillations are indeed *Zitterbewegung*.

[1] Nalitov, A. V., Malpuech, G., Terças, H. & Solnyshkov, D. D. Spin-orbit coupling and the optical spin Hall effect in photonic graphene. *Phys. Rev. Lett.* **114**, 026803 (2015).

[2] Whittaker, C. E. *et al.* Optical analogue of Dresselhaus spin–orbit interaction in photonic graphene. *Nature Photonics* **15**, 193–196 (2020).
